# Compliance to fingolimod and other disease modifying treatments in multiple sclerosis patients, a retrospective cohort study

**DOI:** 10.1186/1471-2377-13-138

**Published:** 2013-10-04

**Authors:** Neetu Agashivala, Ning Wu, Safiya Abouzaid, You Wu, Edward Kim, Luke Boulanger, David W Brandes

**Affiliations:** 1 Novartis Pharmaceuticals Corporation, One Health Plaza, 07936, East Hanover, NJ USA; 2 Evidera, 430 Bedford Street, Suite 300, 02420, Lexington, MA USA; 3 Sweetwater Neurology, 10800 Parkside Drive Suite 202, 37934, Knoxville, TN USA

**Keywords:** Multiple sclerosis, Compliance, Patient adherence, Medication persistence, Discontinuation, Fingolimod

## Abstract

**Background:**

Adherence to disease-modifying therapies (DMTs) results in the reduction of the number and severity of relapses and delays the progression of multiple sclerosis (MS). Patients with lower adherence rates experience more inpatient visits and higher MS-related medical costs. Fingolimod, the first oral DMT approved by the US Food and Drug Administration, may improve the access and compliance to MS treatment when compared to injectable DMTs.

**Methods:**

This retrospective cohort study used pharmacy claims from Medco Health Solutions, Inc., of patients who initiated DMTs between October 2010 and February 2011. Initiation was defined as no prescription fills for the same DMT in the prior 12 months. Patients without a DMT prescription fill 12 months before the index date were considered naïve users. Compliance was measured via proportion of days covered (PDC) and medication possession ratio (MPR) for 12 months post-index. Discontinuation was defined as a ≥60-day gap of index DMT supply. Cox proportional hazard models compared time to discontinuation between cohorts.

**Results:**

Of 1,891 MS patients (mean age: 45.7; female: 76.4%), 13.1% initiated fingolimod, 10.7% interferon beta-1b, 20.0% intramuscular interferon beta-1a, 18.8% subcutaneous interferon beta-1a, and 37.4% glatiramer acetate. Patients initiating fingolimod had highest average PDC and MPR in both experienced (fingolimod: mean PDC=0.83, 73.7% with PDC≥0.8; mean MPR=0.92, 90.5% with MPR≥0.8) and naïve DMT users (fingolimod: mean PDC=0.80, 66.7% with PDC≥0.8; mean MPR=0.90, 87.4% with MPR≥0.8). The proportion of patients discontinuing index DMT within 12 months was significantly lower for the fingolimod cohort (naïve: 31.3%; experienced: 25.7%). Adjusted results found that patients receiving self-injected DMTs discontinued significantly sooner than fingolimod users. This association was generally stronger in experienced DMT users.

**Conclusions:**

Fingolimod initiators were more compliant, less likely to discontinue treatment, and discontinued later than patients who initiated self-injected DMT.

## Background

Multiple sclerosis (MS), a chronic inflammatory disease of the central nervous system, is the most common disabling neurological condition in young people, affecting more than 400,000 individuals in the U.S [1,2]. MS is commonly characterized by intermittent relapses associated with new or worsening neurological symptoms. Age of onset is typically between 20 and 40 years, but diagnosis is often delayed due to its transitory nature and lack of specific diagnostic tests [1]. About 85 percent of those who are newly diagnosed have relapsing MS [2]. Because MS is a lifelong disease with a relatively early onset, lifetime medical costs associated with the disease are substantial [1].

Although there is no known cure for multiple sclerosis, the primary aims of therapy are preventing new relapses, returning function after a relapse, and preventing disability [3]. There are multiple Food and Drug Administration (FDA) approved disease-modifying therapies (DMTs), which are efficacious in reducing the frequency of relapses and/or delaying disability progression [4]. In order to fully benefit from their treatments, patients must follow their prescribed regimen, which may require frequent injections.

Existing studies suggest that compliance to self-injected DMTs is not optimal [5,6]. Portaccio et al. reported 14-44% discontinuation rate of patients who started interferon beta. Common reasons for discontinuation included flu like symptoms and injection site reactions [6]. Needle phobia is another common tolerability issue of injectable medications that may affect treatment continuation. Other factors that may lead to low adherence include depression and anxiety, cognitive impairment, disease progression, or high patient out of pocket copayments [7]. Furthermore, patients may not readily perceive the benefits of such costly, inconvenient, and at times painful treatments since DMTs are prescribed to prevent relatively uncommon but disruptive relapse events and disability progression, which occurs over years.

Fingolimod is the first oral DMT approved by the US Food and Drug Administration. Studies in other disease areas suggest a higher compliance to oral medications than injectable medications [8]. In the absence of the tolerability issues that are known to affect adherence and persistence to the self-injected DMTs, we hypothesized that patients receiving oral fingolimod have a better compliance and a lower discontinuation rate than patients receiving first-line self-injected DMTs.

## Methods

To test our hypothesis, we conducted a retrospective cohort study using pharmacy claims dated between October 2009 and February 2012 from Medco Health Solutions, Inc (Medco). With over 60 million covered lives, Medco is the largest pharmacy benefits management company in the U.S. and has pharmacy claims data for nearly 1 in 5 Americans. The pharmacy claims data from Medco contain national drug codes, prescription details such as days of supply, fill date, and copayment amount, and the characteristics of prescribing physicians as well as dispensing pharmacies. An enrollment file also provides the gender, birth year, start and end dates of pharmacy plan enrollment, and three digit postal zip codes of patient residence.

This study complied with all applicable laws, regulations, and guidance regarding patient protection including patient privacy. All data are compliant to Health Insurance Portability and Accountability Act (HIPPA) and all patient identification is replaced with encrypted random patient identifiers, which can be used to cross link the medical, pharmacy and enrollment files. All the data are stored in a secure server. Only members in the research teams were granted permission by Medco and had access server using assigned credentials. This is a retrospective study analyzing de-identified administrative claims database, so it is exempted from Institutional Review Board (IRB) review.

Patients who initiated one of the first-line DMTs between October 2010 and February 2011 were selected: fingolimod, interferon beta-1b subcutaneous, interferon beta-1a intramuscular, interferon beta-1a subcutaneous, or glatiramer acetate. Medication initiation was defined as no pharmacy claims for the same DMT in the 12 months before the first use dated between October 2010 and February 2011. Patients were then assigned to one of the five DMT cohorts. Fingolimod was the most recently Food and Drug Administration-approved first-line DMT, available in US market since late September, 2010. To obtain sufficient sample size for the fingolimod cohort, patients who initiated fingolimod were included in the fingolimod cohort regardless of prior DMT use. The remaining patients were assigned to one of the four self-injected DMT cohorts based on the first DMT they initiated. The date of the second prescription fill of the index DMT was set as the index date. The rationale for using the second fill date as the index date was that, per FDA recommendation, the first dose of fingolimod is to be administered under physician supervision and with observation for at least six hours following ingestion. Because patients could receive a supply of medication but not start consuming fingolimod until their supervised first dose, any lag in first dose observation would lead to an underestimate of both adherence and persistence measures for fingolimod. Patients who were less than 18 years old and who did not have continuous pharmacy plan enrollment during the 12 months prior to index medication initiation and 12 months following the index date were also excluded.

### Study measures

Demographic characteristics of the selected patients were determined as of the index date including patient age, gender, and geographic region of residence. Patients were classified as DMT naïve patients if they did not have pharmacy claims for any DMT during the year prior to the initiation of the index medication and experienced DMT users if otherwise. Characteristics of the index prescription were also examined. These include the specialty of the prescribing physicians, dosage of the index prescription, copayment amount standardized to a 30-day supply, whether physician needed to obtain prior authorization to prescribe the DMT, whether the DMT was on insurance plan’s formulary, whether the index prescription was filled via mail-in order, and type of pharmacy that dispensed the DMT. Dispensing pharmacies were classified into three types: Accredo Health Group, Inc, other specialty pharmacies, and retail pharmacies. Accredo Health Group, Inc is a specialty pharmacy that distributes medications and provides associated services to patients with complex and chronic diseases through Therapeutic Resource Centers.

**Figure 1:**
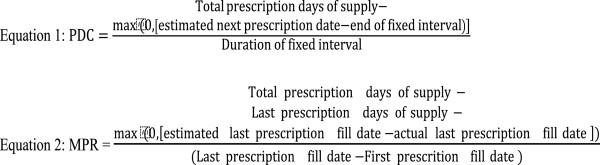
Adherence Equations.

Adherence to index DMT was assessed based on the pharmacy claims dated within 12 months following the index date (Figure 1). Proportion of days covered (PDC) was measured as the total number of days with medication supply in the 12-months follow-up period divided by 365 days (Equation 1). Medication possession ratio (MPR) was estimated as total days with medication supply within the refill interval divided by number of days in the refill interval (Equation 2). Extra days of medication supply that covered the periods after the end of 12-month follow-up were deducted from the numerator of both PDC and MPR measures. Time to discontinuation was defined as the number of days to the beginning of the first 60-day gap in supply of index medication. The 12-month persistence rate was measured as the proportion of patients who did not discontinue the index DMT during the 12 months since their initiation. We assessed the distribution of PDC, MPR and persistence measures as continuous measures, and created ordinal categorical measures (e.g., 0≤PDC<0.2, 0.2≤PDC<0.4, 0.4≤PDC<0.6, 0.6≤PDC<0.8 and 0.8≤PDC≤1) and reported the proportion of patients in each category.

### Data analysis

The distribution of baseline characteristics and outcomes was assessed for each of the DMT cohort using percentages for categorical variables, and mean and standard deviation (SD) for continuous measures. Using the fingolimod cohort as the reference group, unadjusted comparisons were conducted between the fingolimod cohort and each of the remaining cohorts using Chi-square tests for categorical variables and Student *t*-tests for continuous variables. Non-parametric Wilcoxon rank-sum tests were used for count variables and copayment amount. The assessment of the distribution of compliance measures were further stratified by patients’ prior experience with DMT (i.e., naïve vs. experienced).

Time to discontinuing index DMT was estimated and compared between cohorts. Kaplan-Meier survival curves were plotted for DMT naïve and experienced users, respectively. Cox proportional hazard models were estimated to identify factors associated with time to index DMT discontinuation using demographic and index prescription characteristics as covariates. Separate Cox proportional hazard models were estimated for naïve and experienced DMT users.

## Results

Of the 1,891 DMT initiators identified, 13.1% initiated fingolimod, 10.7% interferon beta-1b, 20.0% intramuscular interferon beta-1a, 18.8% subcutaneous interferon beta-1a, and 37.4% glatiramer acetate (Table 1). Mean (SD) age was 45.6 (11.1) years, and 76.4% were females. Overall, 75.4% of DMT initiators were naïve users. A significantly lower percentage of users of fingolimod (38.7%) were naïve to DMT treatment than the other cohorts (72.4-83.7%, Table 1).

**Table 1:** Demographic and index prescription characteristics

	**Fingolimod**	**Interferon beta-1b**		**Intramuscular interferon beta-1a**		**Subcutaneous interferon beta-1a**		**Glatiramer acetate**	
	**N=248**	**N=202**		**N=379**		**N=355**		**N=707**	
			p-value		p-value		p-value		p-value
Age Categories (%)			*0.3112*		*0.1516*		*0.0001*		*0.1733*
18-24	3.2	2.5		3.4		2.0		1.8	
25-34	10.5	17.8		13.5		19.2		15.6	
35-44	24.6	23.3		28.0		33.2		27.4	
45-54	37.5	33.7		27.2		31.6		33.1	
55-64	21.0	18.3		22.4		10.1		17.8	
65-74	3.2	4.5		5.0		3.9		3.7	
75-84	0	0		0		0		0.6	
Age: mean (SD)	46.4 (10.7)	45.5 (11.2)	*0.4107*	46.5 (11.5)	*0.9349*	43.5 (10.7)	*0.0012*	45.9 (11.1)	*0.5079*
Female (%)	79.0	70.3	*0.0330*	79.7	*0.8437*	74.4	*0.1850*	76.4	*0.3925*
Region (%)			*0.2046*		*0.0008*		*0.0984*		*0.0126*
Northeast	18.2	17.3		31.4		23.7		29.1	
Midwest	29.4	27.7		28.2		33.0		27.2	
South	32.7	41.1		28.2		26.8		27.3	
West	19.8	13.9		12.1		15.8		16.1	
Other/missing	0.0	0.0		0.0		0.9		0.3	
Naïve to DMT treatment (%)	38.7	82.7	*<0.0001*	82.6	*<0.0001*	72.4	*<0.0001*	83.7	*<0.0001*
Specialty of prescriber: Neurologist (%)	82.3	78.2	*0.2825*	74.7	*0.0257*	74.7	*0.0270*	77.5	*0.1159*
Index medication days supply >= 90 days (%)	0.0	0.5	*0.2673*	0.0	*NA*	0.6	*0.2364*	13.6	*<0.0001*
Prior authorization (yes) (%)	56.1	60.4	*0.3527*	48.6	*0.0662*	46.2	*0.0173*	47.4	*0.0189*
DMT on formulary (yes) (%)	15.3	71.8	*<0.0001*	97.9	*<0.0001*	85.1	*<0.0001*	96.2	*<0.0001*
Mail order (%)	74.6	51.5	*<0.0001*	47.0	*<0.0001*	54.1	*<0.0001*	54.0	*<0.0001*
Days’ Supply: mean (SD)	33.3 (16.3)	35.7 (19.5)	*0.1709*	35.2 (18.2)	*0.2040*	35.0 (18.4)	*0.2302*	38.8 (21.4)	*<0.0001*
30 day Co-pay: mean (SD)	98.5 (315.5)	101.9 (291.0)	*0.9063*	124.1 (376.1)	*0.3588*	88.4 (255.6)	*0.6768*	119.2 (333.5)	*0.3944*

P-values based on comparison with fingolimod cohort using Chi-square tests for categorical variables and Student's *t*-test for age, and Wilcoxon rank-sum tests were used for days' supply and copayment amount.

DMT: disease-modifying therapy. SD: standard deviation.

A higher percentage of fingolimod was prescribed by neurologists compared to other DMT cohorts (Table 1). While no users of fingolimod received an index medication supply for 90 days or more, 13.6% of glatiramer acetate users did receive at least a 90-day of supply (p<0.0001). Mail order prescriptions were utilized significantly more frequently for fingolimod users (74.6%) than all other DMT users (all p<0.0001). The mean co-payment for a 30-day supply of fingolimod was $98.5 (SD: $315.5), which was not significantly different from other DMT cohorts.

Among naïve DMT users, the fingolimod cohort had a significantly higher PDC (mean: 0.80, SD: 0.23) and MPR (mean: 0.90, SD: 0.09) than the other four DMT cohorts (Table 2). Similarly, fingolimod users had a significantly higher proportion of patients included in the most compliant category range. For example, 87.4% of fingolimod cohort had MPR≥0.8, higher than the four injectable DMT cohorts (range 72.8% to 81.5%). A significantly higher percentage of fingolimod users (68.8%) were persistent at 12 months compared to other cohorts (range 46.1% to 56.3%, all p<0.05). Similar patterns were observed in experienced DMT users, but the between-cohort difference favoring fingolimod was larger (Table 3).

**Table 2:** Compliance and persistence measures of users naïve to disease-modifying agents

	**Fingolimod**	**Interferon beta-1b**		**Intramuscular interferon beta-1a**		**Subcutaneous interferon beta-1a**		**Glatiramer acetate**	
	**N=96**	**N=167**		**N=313**		**N=257**		**N=592**	
			p-value		p-value		p-value		p-value
PDC: mean (SD)	0.80 (0.23)	0.65 (0.31)	*<0.0001*	0.72 (0.30)	*0.0027*	0.67 (0.31)	*<0.0001*	0.72 (0.29)	*0.0017*
PDC Categories (%)			*0.0003*		*0.0347*		*0.0043*		*0.0127*
0-<0.2	3.13	11.38		8.63		13.62		6.93	
0.2-<0.4	7.29	16.77		10.86		12.45		11.49	
0.4-<0.6	4.17	12.57		10.54		8.95		13.51	
0.6-<0.8	18.75	17.37		18.53		15.56		14.02	
0.8-1	66.67	41.92		51.44		49.42		54.05	
MPR: mean (SD)	0.90 (0.09)	0.86 (0.17)	*0.0055*	0.88 (0.16)	*0.0502*	0.88 (0.15)	*0.0551*	0.89 (0.15)	*0.1769*
MPR Categories (%)			*0.0349*		*0.1255*		*0.3037*		*0.2619*
0-<0.2	0.00	0.62		0.68		0.41		0.36	
0.2-<0.4	0.00	1.85		1.69		1.24		1.79	
0.4-<0.6	1.05	8.02		6.78		4.96		5.02	
0.6-<0.8	11.58	16.67		11.53		13.22		11.29	
0.8-1	87.37	72.84		79.32		80.17		81.54	
Persistence (<60 day gap) (%)	68.75	46.11	*0.0004*	53.67	*0.0090*	55.25	*0.0218*	56.25	*0.0213*
Persistent days (days till first 60-day gap) (%)									
No gap	68.75	46.11		53.67		55.25		56.25	
1-90	6.25	16.77		14.38		18.68		13.68	
91-180	9.38	14.37		10.22		12.06		11.82	
181-270	8.33	14.37		13.74		8.56		10.98	
270+	7.29	8.38		7.99		5.45		7.26	
Switched to other DMTs after discontinuation (%)	1.04	13.17	*0.0008*	7.99	*0.0147*	11.67	*0.0017*	7.77	*0.0153*

P-values based on comparison with fingolimod cohort, using Chi-square tests for categorical variables and Student’s *t*-tests for continuous variables.

DMT: disease-modifying therapy. SD: standard deviation. PDC: proportion of days covered. MPR: medication procession ratio.

**Table 3:** Compliance and persistence measures of users experienced to disease-modifying agents

	**Fingolimod**	**Interferon beta-1b**		**Intramuscular interferon beta-1a**		**Subcutaneous interferon beta-1a**		**Glatiramer acetate**	
	**N=152**	**N=35**		**N=66**		**N=98**		**N=115**	
			p-value		p-value		p-value		p-value
PDC: mean (SD)	0.83 (0.23)	0.61 (0.30)	*0.0002*	0.67 (0.32)	*0.0004*	0.68 (0.31)	*0.0001*	0.73 (0.30)	*0.0040*
PDC Categories (%)									
0-<0.2	4.61	17.14	*0.0009*	9.09	*0.0002*	10.20	*0.0002*	6.09	*0.0003*
0.2-<0.4	3.95	8.57		22.73		11.22		11.30	
0.4-<0.6	2.63	11.43		4.55		12.24		13.91	
0.6-<0.8	15.13	22.86		10.61		18.37		7.83	
0.8-1	73.68	40.00		53.03		47.96		60.87	
MPR: mean (SD)	0.92 (0.08)	0.82 (0.18)	*0.0066*	0.88 (0.13)	*0.0287*	0.84 (0.19)	*0.0004*	0.89 (0.16)	*0.0602*
MPR Categories (%)			*0.0001*		*0.0069*		*0.0004*		*0.0106*
0-<0.2	0.00	0.00		0.00		1.10		0.93	
0.2-<0.4	0.00	3.33		0.00		3.30		1.85	
0.4-<0.6	0.00	10.00		5.00		5.49		5.56	
0.6-<0.8	9.52	16.67		16.67		18.68		6.48	
0.8-1	90.48	70.00		78.33		71.43		85.19	
Persistence (<60 day gap) (%)	74.34	42.86	*0.0003*	53.03	*0.0020*	54.08	*0.0009*	62.61	*0.0396*
Persistent days (days till first 60-day gap) (%)									
No gap	74.34	42.86		53.03		54.08		62.61	
1-90	6.58	22.86		15.15		19.39		12.17	
91-180	4.61	5.71		21.21		10.20		13.91	
181-270	9.21	17.14		6.06		10.20		6.96	
270+	5.26	11.43		4.55		6.12		4.35	
Switched to other DMTs after discontinuation (%)	4.61	11.43	*0.1219*	13.64	*0.0188*	9.18	*0.1488*	13.04	*0.0130*

P-values based on comparison with fingolimod cohort, using Chi-square tests for categorical variables and Student’s t-tests for continuous variables.

DMT: disease-modifying therapy. SD: standard deviation. PDC: proportion of days covered. MPR: medication procession ratio.

**Figure 2:**
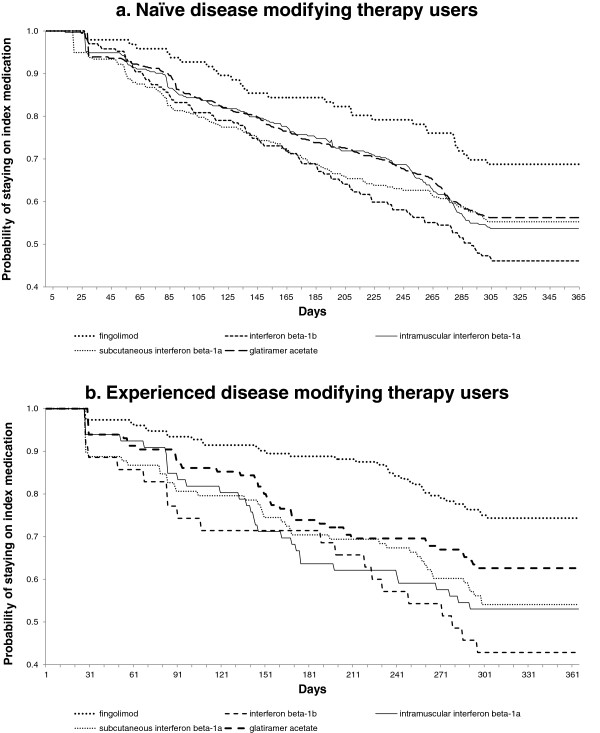
Time to discontinuation of initiated desease-modifying agent.

Figures 2a and 2b depict the unadjusted time to discontinuation of initiated disease-modifying agents for naïve and experienced DMT users. In both cases the fingolimod cohort had higher medication persistence over the observation period.

Results from the Cox proportional hazard models demonstrated that patients who initiated self-injected DMTs had a higher hazard of discontinuing index medication than patients who initiated fingolimod in both experienced and naïve DMT users (Table 4). The association was stronger in experienced users than in naive users.

**Table 4:** Time to index DMT discontinuation: cox proportional hazard models for experienced and naïve DMT users

	**Experienced DMT Users**		**Naïve DMT Users**	
	**Hazard ratio**	**95% Confidence interval**	**Hazard ratio**	**95% Confidence interval**
DMT initiated				
Fingolimod	Reference		Reference	
Interferon beta-1b	**2.83**	**(1.62-4.93)**	**1.90**	**(1.25-2.89)**
Intramuscular interferon beta-1a	**2.43**	**(1.49-3.97)**	**1.53**	**(1.03-2.28)**
Subcutaneous interferon beta-1a	**2.42**	**(1.54-3.82)**	**1.58**	**(1.05-2.38)**
Glatiramer acetate	**1.73**	**(1.11-2.69)**	**1.53**	**(1.04-2.24)**
Gender				
Male	Reference		Reference	
Female	1.45	(0.99-2.11)	1.07	(0.89-1.29)
Age categories				
18-44	Reference		Reference	
45-54	0.78	(0.55-1.11)	**0.81**	**(0.68-0.98)**
55-64	0.97	(0.65-1.46)	**0.75**	**(0.60-0.94)**
65+	0.80	(0.38-1.69)	0.67	(0.44-1.01)
Region				
West	Reference		Reference	
Northeast	0.68	(0.40-1.15)	0.78	(0.60-1.01)
Midwest	0.99	(0.62-1.59)	0.88	(0.69-1.13)
South	1.19	(0.75 -1.87)	0.88	(0.69-1.12)
Prior authorization for index prescription	1.21	(0.88-1.65)	**1.25**	**(1.06-1.48)**
Copayment for index prescription				
0-<30	Reference		Reference	
30-100	0.93	(0.65-1.32)	0.95	(0.78-1.15)
100+	0.92	(0.59-1.45)	0.88	(0.70-1.09)
Mail order for index prescription	1.10	(0.59-2.07)	**0.57**	**(0.42-0.78)**
Pharmacy type for index prescription				
Retail	Reference		Reference	
Accredo	1.03	(0.54 -1.97)	1.05	(0.77-1.43)
Other specialty pharmacy	1.69	(0.86-3.31)	0.78	(0.56-1.07)

Bold numbers indicate that the hazard ratios are significantly different from 1 at p<0.05.

## Discussion

To date, there have been limited publications comparing compliance and persistence to DMT treatments among patients with multiple sclerosis [6,9,10]. Using pharmacy claims, our study assessed the adherence and persistence to first-line DMT treatments shortly after the approval of the first oral DMT in the US. Similar to other recent studies of individuals with MS, the patients included in our study were predominantly female with a mean age in the 40s [9,10]. About three quarters of the MS patients in our study were naïve to DMT treatment, though a significantly lower proportion of fingolimod users (38.7%) were naïve to DMT treatment than the self-injected DMT users. A likely reason for this observation is that fingolimod’s recent availability prompted patients to switch from their existing DMTs, potentially seeking a more efficacious or tolerable treatment alternative. Compared to the compliance and persistence measures of patients who had failed at least one DMT reported by Halpern et al [9], the MPR and proportion of persistent users among the experienced DMT users in our study was generally higher. The differences in MPR values may be because that Halpern followed patients for a longer period (averaged between 1.1-1.3 years across DMT cohorts) and used a slightly different method for estimating MPR. Halpern et al defined MPR as “… days supply of the second-line DMT divided by days on second line DMT” which did not contain sufficient details for comparing results across studies. The method used for this study deducts the excess supply of the DMT at the end of the follow-up period.

Our study found that patients initiating fingolimod were more compliant and persistent to their treatment than patients initiating self-injected DMTs. One probable explanation is that patients may find it easier to adhere to oral treatments with fewer side effects than injectable DMTs associated with flu-like symptoms and injection site reactions. Both flu-like symptoms and injection site reactions were among the top reasons for discontinuing the treatment with interferon beta in previous studies [11]. Other commonly cited reasons included forgetting to take the medication and depression. Based on an online survey of patients using self-injected DMTs, Treadaway et al also found that, among patients who missed at least one dose, 32% reported injection-related reasons, such as not feeling like taking injections (22%), tired of taking injection (17%), pain at injection site (7%), and the absence of someone to help administer the medication (4%) [12]. Patients who missed >25% of their doses were more likely to report injection anxiety (11% vs. 1%) and not feeling like taking injections (29% vs. 20%) than those who missed less doses [12]. This suggests that intolerance of self-injected DMTs, among all the factors that affect patient treatment adherence, plays an important role in “true” non-adherent users.

The association between poor compliance and treatment intolerance and side effects is not unique in patients with multiple sclerosis. Such association has been identified in various chronic disease areas which require long-term treatments, such as HIV antiretroviral therapy, as well as in oral treatments with gastrointestinal tolerability issues shown among cancer patients [13-15]. In addition to tolerability issues and side effects of the treatments, self-injection is often associated with additional barriers to compliance and persistence, including storage issues and priming needles [13]. The availability of new oral DMTs will provide an alternative for MS patients who cannot tolerate the self-injected DMTs and improve adherence to DMT treatment.

Despite the better treatment compliance observed in patients initiating fingolimod than other DMT cohorts, 27.8% of fingolimod users discontinued the medication within 1 year since the treatment initiation (25.7% among experienced DMT users and 31.3% among naïve DMT users). While oral medications may offer an improvement in patient compliance to DMT treatment, other strategies or programs may need to be tested and implemented to further improve the treatment adherence for patients with chronic illness.

Early treatment and adherence to DMT treatment are two key factors for the effective prevention of relapses and disability. Sormani et al reported that treatments that reduce relapses during the first two years of having the disease are associated with a lower accumulation of neurological disability [16]. Using real-world data, Tan et al demonstrated the association between DMT adherence and patient clinical and economic outcomes. Tan et al found that being adherent to DMTs (defined as MPR≥80%) was associated with a 37% lower risk of having an MS-related hospital admission, a 29% lower risk of having an MS relapse, and about $1,000 less in healthcare costs during the 12-month follow-up period [17]. Steinberg’s research found similar results with adherent patients tending to have a lower risk of inpatient admission over a 3 year period [7,17]. With fewer relapses and hospitalizations, patients are more able to experience an improved quality of life as well as reduced adverse effects. For this reason, selecting treatments that are highly efficacious and tolerable will maximize the likelihood of optimal management of MS relapse activity and long-term outcomes.

This study has several limitations. We might not have captured all the eligible patients from the Medco database because only pharmacy claims were used to identify MS patients. In addition, we did not measure the outcomes associated with different DMTs, or the clinical and economic consequences of differences in medication adherence and persistence. Such a study would require a longer follow-up time and a larger sample size, neither of which were possible due to the recent availability of fingolimod. Additionally, patients were considered DMT naïve if they did not have DMT prescription fill during 12 months prior to the index date. Some experienced DMT users may have been misclassified as naïve DMT uses if they stopped DMTs for 1 year or longer prior to the index date for different reasons such as pregnancy, breast feeding, non-compliance, or enrolled in clinical trials of DMT. The association between fingolimod use and treatment compliance was in the same direction among both experienced and naïve DMT users, therefore, it is unlikely that the misclassification has affected the study conclusion. The analyses were also limited by the variables in the database. For the majority of the patients, we used pharmacy claims only to infer that patients were taking oral medications to treat MS; Compliance to DMT was assessed based on dispensed medications rather than medications consumed by the patients. Further studies using patient-reported compliance may provide a more accurate estimate of patient compliance. Further event-based studies are warranted as data become available. Finally, while we adjusted for available demographic and other covariates in our models, unobserved factors may have contributed to differences in adherence and persistence.

## Conclusion

The oral MS treatment fingolimod is associated with lower rates of discontinuation and higher rates of adherence in comparison to self-injected DMTs. One potential rationale is the improved tolerability of fingolimod. The emergence of other oral DMTs with differing tolerability profiles will help clarify this issue of tolerability versus formulation as a mediator of adherence and persistence.

## Abbreviations

FDA: Food and drug administration; DMTs: Disease-modifying therapies; MPR: Medication possession ratio; MS: Multiple sclerosis; PDC: Proportion of days covered; SD: Standard deviation.

## Competing interests

The authors declare that they have no competing interests.

## Authors’ contributions

NA, SA and EK developed study concept and collaborated in development of study design, and manuscript, and conducted a critical review. NW, YW, LB developed study design, conducted analysis, and wrote manuscript. DB completed interpretation and conducted critical review of manuscript. All authors read and approved the final manuscript.

## Pre-publication history

The pre-publication history for this paper can be accessed here:

http://www.biomedcentral.com/1471-2377/13/138/prepub
